# Electric Discharge Machining of Ti6Al4V ELI in Biomedical Industry: Parametric Analysis of Surface Functionalization and Tribological Characterization

**DOI:** 10.3390/ma16124458

**Published:** 2023-06-19

**Authors:** Muhammad Umar Farooq, Saqib Anwar, Haider Ali Bhatti, M. Saravana Kumar, Muhammad Asad Ali, Muhammad Imam Ammarullah

**Affiliations:** 1 School of Mechanical Engineering, University of Leeds, Leeds LS2 9JT, UK; 2 Industrial Engineering Department, College of Engineering, King Saud University, P.O. Box 800, Riyadh 11421, Saudi Arabia; 3 Department of Industrial and Manufacturing Engineering, University of Engineering and Technology, Lahore 54890, Pakistan; 4 Graduate Institute of Manufacturing Technology, National Taipei University of Technology, Taipei 10608, Taiwan; 5 Department of Mechanical Engineering, Faculty of Engineering, Universitas Pasundan, Bandung 40153, West Java, Indonesia

**Keywords:** titanium alloy, Ti6Al4V, electrical discharge machining, surface topography, parametric analysis, tribology

## Abstract

The superior engineering properties and excellent biocompatibility of titanium alloy (Ti6Al4V) stimulate applications in biomedical industries. Electric discharge machining, a widely used process in advanced applications, is an attractive option that simultaneously offers machining and surface modification. In this study, a comprehensive list of roughening levels of process variables such as pulse current, pulse ON time, pulse OFF time, and polarity, along with four tool electrodes of graphite, copper, brass, and aluminum are evaluated (against two experimentation phases) using a SiC powder-mixed dielectric. The process is modeled using the adaptive neural fuzzy inference system (ANFIS) to produce surfaces with relatively low roughness. A thorough parametric, microscopical, and tribological analysis campaign is established to explore the physical science of the process. For the case of the surface generated through aluminum, a minimum friction force of ~25 N is observed compared with the other surfaces. The analysis of variance shows that the electrode material (32.65%) is found to be significant for the material removal rate, and the pulse ON time (32.15%) is found to be significant for arithmetic roughness. The increase in pulse current to 14 A shows that the roughness increased to ~4.6 µm with a 33% rise using the aluminum electrode. The increase in pulse ON time from 50 µs to 125 µs using the graphite tool resulted in a rise in roughness from ~4.5 µm to ~5.3 µm, showing a 17% rise.

## 1. Introduction

Titanium and its derivatives are multifaceted, encompassing the biomedical industries (due to their uses in orthopedic and orthodontic implants) [1]. The most popular alloy, Ti6Al4V, is frequently used because of its high strength-to-weight ratio, high fatigue and loading strength, high corrosion resistance, biocompatibility, and retention of mechanical properties in elevated temperatures. These properties of the alloy make it durable and sustainable in various specialized applications [2]. Along with these desirable properties, there are limitations such as lower thermal conductivity, a small modulus of elasticity, less reactivity, and a large strength-to-weight ratio, which reduce its machineability and result in different challenges for processing [3,4]. Among processing techniques, various conventional machining methods have reduced its applications because of limitations such as the production of complex profiles, accurate and precise machining, improved surface integrity, and low primary and secondary processing costs, which make it difficult to choose hard-to-cut materials [5]. Considering the above limitations, nonconventional machining processes are considered, which provide a solution to ease the boundaries of conventional machining. Among these processes, electric discharge machining is the leading-edge machining process used to machine hard-to-cut materials where there is a requirement to machine an intricate geometrical shape with greater dimensional accuracy. This process utilizes thermoelectric energy to erode conductive workpieces through rapidly recurring electric sparks. During the process, no direct contact exists between the tool electrode and the workpiece; therefore, no residual stresses are produced. The process requires the tool and workpiece to be immersed in a dielectric liquid [6]. The machined surface contains random features such as craters and porosity, and has improved mechanical properties because of the surface treatment [7,8,9,10].

The electric discharge machining process is stochastic and complex, requiring significant control over parameters to optimize performance. This complexity requires researchers to investigate the effects of various input responses on machining materials with different properties. The machining of Ti alloy in powder-mixed dielectric generates a surface that carries a higher probability of attracting osteoblast cells because of its rougher features [11]. The surfaces with these features are surrounded by water molecules and absorbed ions in the blood. In these cases, a layer of TiO_2_ generates a protective layer that creates a barrier between the implant surface and the molecules, and corrosion is inhibited at this stage. Considering the benefits on the application side, the limitation of the EDM process is its low productivity, which is strongly linked to surface integrity (if one objective is approached, the other deteriorates). A literature survey reveals various studies in which researchers have tried to improve machining efficiency regarding the material removal rate (MRR) while sustaining the surface attributes. Various parametric-analysis-based approaches have been used to improve the process conditions. For instance, Ehsan et al. [12] used grey relational analysis, Rafaqat et al. [13] used objective compromise, Sharma et al. [14] used ANFIS, and Ishfaq et al. [15] used the desirability function. Recently, much focus has been shifted towards black box models, which accurately model complex processes with multiphysics problems. In this regard, the artificial neural network (ANN) is an intelligent information-processing model that has been utilized to develop a predictive model and analysis in manufacturing [14]. An adaptive neural fuzzy inference system (ANFIS) is a hybrid intelligent system that obtains specific data and develops a prediction model. Sharma et al. [14] used ANFIS for modeling the electric discharge machining process during the machining of Inconel 625 and carried out optimization using response surface methodology. Tang and Du [16] used tap water to carry out electric discharge machining of Ti6Al4V. The authors used duty factor, polarity, lift height, voltage, and current to evaluate roughness using a Taguchi orthogonal array. Similarly, Hassanin et al. [17] used selective laser-sintered Ti6Al4V as an implant material and two modes of electric discharge machining, specifically roughening and finishing. The process was modeled using central composite design regression. Tiwary et al. [18] evaluated Cu-mixed deionized water during microimpression machining on Ti6Al4V using variable pulse currents. The authors discussed the fact that control over energy transfer is very important in determining the final quality of the surface. Sultan et al. [19] employed pulse OFF time as an input electrical parameter to machine EN 353 steel. The authors reported that a balanced pulse OFF time helps produce a better surface finish and a higher material removal rate. In addition to electrical parameters, electrode materials were chosen to reduce the tool wear with a higher material removal rate. However, for tribological applications in which parts slide on each other, causing friction and wear, choosing a suitable tool material that gives a better surface finish can help increase the lifespan of the machined parts. In addition, the dielectric medium can also be altered to facilitate the process of achieving improved surface properties. Dong et al. [20] utilized a graphite tool in a water/oil emulsion and pure kerosene. The authors achieved minimum tool wear and a maximum MRR with fewer surface cracks on the machine surface with the modified dielectric. Payal et al. [21] have investigated the machinability of ENI tool steel using copper, brass, and graphite electrodes. The authors compared the machined surfaces and heat-affected zones based on thermal and electrical conductivity, the melting point, and the density of the electrodes. Theo et al. [22] discussed the spongy and spherical texture produced during electric discharge grinding when a material is processed using aluminum electrodes. Compared with negative polarity, positive polarity produced higher surface roughness and significantly determined material removal and surface integrity. The redeposited layer comprised dielectric breakdown products and pyrolysis-induced material migration from the tool and the workpiece [8]. Thermal loading, cyclic heating, and residual strains influence a material’s key characteristics, and only post-finishing/processing procedures may alleviate these factors. The process settings and operating conditions determine the thickness of the reformed layer. For instance, the dielectric effect resulted in substantial carbon diffusion into the reformed layer during its production and solidification in the case of hydrocarbon as compared with deionized water [15]. Moreover, Ti6Al4V can generate thick oxide layers which merge with the recast layer formed during machining, providing an extra benefit for developing new surface layers. Carbon diffusion and electrode material migration result in a high level of hardness, which improves the surface integrity of Ti6Al4V surfaces. Similarly, other surface characteristics must be investigated to improve tribological applications, increase wear and corrosion resistance, and reduce friction loss, energy loss, and cytotoxicity in biological implants [22]. A machined surface with more cracks, pores, and voids tends to increase friction due to shear forces, which originate wear and energy losses. Different sintered tool materials and additives are used to uniformly distribute the carbon, which influences the cracks and voids. The tool material significantly influences the surface properties by providing inherent lubrication. This type of surface helps reduce shear force and energy losses by improving tribological properties [1]. Rajurkar et al. [23] evaluated surface craters and cracks using electrophysical and chemical processes during nano machining and reported the surface texture based on the arithmetic mean. Bui et al. [24] evaluated the modified surfaces of titanium implants during powder-mixed discharge machining. They highlighted the need to evaluate other surface characteristics, such as peak-to-valley details, as implants have tribological applications.

On the other hand, the tribological properties of Ti6Al4V have been extensively demonstrated to be limited in the domains of artificial hip and knee joint implantations, pumps, valves, bearings, and other similar applications. The underlying reasons for this include excessive friction and wear, corrosion (galvanic and crevice), and material embrittlement, which results in material seizing and damage [1,7]. Plasma nitriding, plasma electrolytic oxidation, physical vapor deposition (PVD), chemical vapor deposition (CVD), ion implantation, and laser surface texturing have all been used to improve the tribological properties of Ti alloys. The techniques indicated above were forced to be reserved for specialized applications due to their uneconomic nature and inability to build massive protective layers [8]. The surface interaction of Ti6Al4V with the counter body during tribological applications can cause significant contact stresses, necessitating robust protection coatings. According to the literature, the passivation effect of functional material surfaces can be achieved with thermal oxidation (ThO). The adherent oxide layer development and surface/diffusion hardening had the most consistent influence on the process at high temperatures [7]. However, electric discharge machining can generate carbides and oxides on the surface, which improves surface properties by several folds. Mughal et al. [9] showed the enhanced surface hardness of Ti6Al4V ELI grade 23 because of the formation of carbides and oxides. Similarly, Al-Amin et al. [10] found amorphous and crystalline phases of carbides and oxides on the machined surface of 316L. The authors showed an improved surface with reduced crack formation and enhanced morphological features, such as nanopores and shallow craters. Thus, the process is capable of simultaneously machining as well as modifying the surface.

The published literature identifies that Ti6Al4V possesses excellent mechanical and engineering attributes in various applications. However, being a hard-to-cut material, its machining is challenging considering the industrial use cases. In this regard, the machining challenges associated with the material are thoroughly explored in this research, which considers a wide range of process parameters such as pulse current, pulse ON time, pulse OFF time, and polarity. A SiC-mixed dielectric was used based on the recommendations of Farooq et al. [6] for machining titanium alloy. Similarly, Mughal et al. [8] recommended using a SiC-mixed dielectric for improved surface integrity and related attributes. Li et al. [25] showed the supremacy of the SiC-mixed EDM process in achieving enhanced surface characteristics as compared with the normal dielectric. However, the efficiency of the process concerning tribological applications has not been comprehensively explored because of the higher numbers of surface defects. Therefore, a thorough investigation is needed, and is carried out herein, along with a consideration of several process variables. Considering tribological applications, a range of electrode materials is also explored as a variable that influences surface features. To summarize, the novelty of this work lies in its evaluation of surface modification and functionalization assessment.

There are substantial points that were identified to be addressed in the current study.

- There is no evidence of the adaptive neural fuzzy inference system approach being applied to SiC-mixed electric discharge machining processes while employing different tool materials.
- No comprehensive studies are available on the tribological performance of surfaces produced through SiC-mixed electric discharge machining while employing different tool materials.
- The current study extends the work carried out by Farooq et al. [6], using a wider range of electrodes, process modeling, and tribological characterization.

The machinability of titanium alloys is not as mature as that of steels, so it requires comprehensive evaluation employing roughening parameters. It is pertinent to highlight the need for a wide-ranging process analysis based on surface characteristics such as roughness parameters, morphology, and layer properties. Moreover, the adaptive neural fuzzy inference system approach is used to model the process response. The experimentation results are thoroughly studied through parametric control, morphological, and tribological analyses. A detailed explanation of the physical phenomena of the process is compiled in line with the results from different dimensions.

## 2. Materials and Methods

The workpiece material Ti6Al4V (ELI) was employed for the experimentation. The square plate of the workpiece, which had 100 × 100 × 4 mm^3^ dimensions, was used. The material was chosen because of its wide applications in biomedical industries. In the biomedical industry, several implants have the chosen material as a substrate because of its toughness and other engineering attributes [2]. The physical attributes of Ti6Al4V that potentially affect thermoelectrical erosion process are mentioned in Table 1 based on the details provided by the manufacturer (BaoJi Titanium Industry Co., Ltd., BaoJi, China).

The selection of the process conditions which result in higher efficiency is the objective of this study. The process is highly dependent on the thermal and electrical properties of materials (tool materials and workpieces) involved in machining. Ahmed et al. [27] evaluated the potentiality of microimpressions on Ti6Al4V using Al, Cu, Br, and Gr as tool electrodes. The authors discussed the effect of material properties on the process dynamics. The authors commented that the properties of Ti6Al4V are not straightforwardly predictable compared with those of steels, and this necessitates evaluating a range of materials as tool electrodes. Therefore, a range of material combinations needs to be tested. Tool electrode selection is not as simple for Ti6Al4V as it is for other materials, such as steel, which is reasonably matured. The possible reasons behind this uncertainty are the mechanisms involved at the tool–workpiece interface during machining (vaporization, melt dynamics, and evacuation of debris). For this reason, four electrodes, including copper (Cu), brass (Br), graphite (Gr), and aluminum (Al) electrodes, were used to machine the workpiece, each having a 15 mm diameter. Similarly, Raza et al. [28] discussed the influence of material properties on processing conditions, motivating the choice of four electrode materials. The physical properties of tool electrodes which influence the process are mentioned in Table 2.

**Figure 1:**
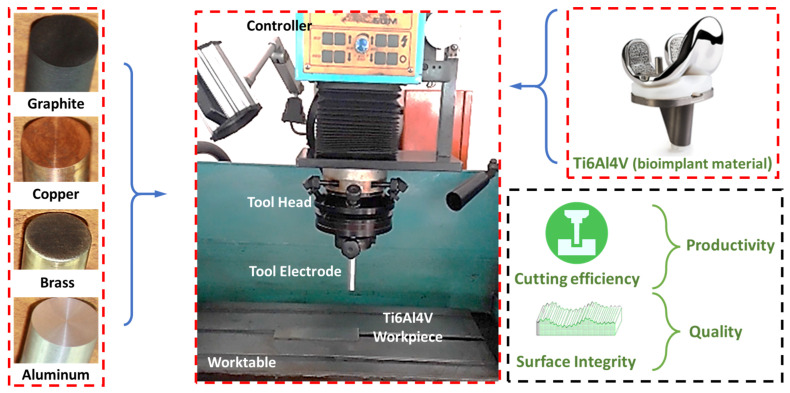
Experimental setup illustration: an overview of tool electrodes used for experimentation, workpiece, and key measures.

A dedicated machine tool for the whole experimentation process, CNC EDM die-sinker machine (RJ—230 Creator: Taiwan), was used. An overall graphical representation of the methodology is shown in Figure 1, where four tool electrodes are indicated with the machine tool. A circular cavity of 300 µm in depth was machined in each experiment. The dielectric medium, commercially available kerosene oil with 5 g/L SiC concentration, was used during machining. The diameter (70–80 μm), density (3.1 g/cm^3^), and thermal conductivity (120 W/m-K) are considered necessary to know during the machining action at the interface of the tool and workpiece. As reported in the literature by Li et al. [25], the use of abrasive SiC powder in dielectric results in superior surface attributes such as a controlled white layer, low roughness, and improved hardness. Similarly, Mughal et al. [8] evaluated different SiC powder concentrations in kerosene and their performance on surface quality. Farooq et al. [6] recommended the use of particular levels of powder concentrations for improved machinability. The physical properties of the SiC powder affect the process of decreasing the number of surface defects. As per Li et al. [25], the powders’ thermal conductivity and electrical resistivity change the dielectric properties, which facilitates balanced plasma channel generation. The experimentation was carried out in two phases; the first included preliminary experiments without a design of experiments, and the second involved mature experiments under the design of experiments.

In the first phase, the selection of significant parameters based on the literature and the suitability of those variable values were searched out. The selection was made based on criteria including excessive sparking, interruption, incomplete machining, and improper mixing of the added powder. Therefore, a range was selected to ensure adequate quality of the machined part, balanced distribution of the sparks, and no or minimal traces of burning.

The process variables which were selected for the mature experimentation phase were electrode material (El), pulse current (PC), pulse ON time (ON), pulse OFF time (OFF), and polarity (PO), and their levels are mentioned in Table 3. The pulse current is directly related to the energy transfer to the workpiece. After extensive trial experiments, the roughening levels were considered to carry out predictive analysis in extreme processing conditions. The specific levels were chosen on the guidelines of Ahmed et al. [27] and Ishfaq et al. [15]. Sultan et al. [19] commented on the mechanistic influence of pulse ON time. The particular range of the pulse OFF time was based on guidelines from Mughal et al. [8] and trial experimentation. Ehsan et al. [12] evaluated and supported the need for a sufficient window of pulse OFF time. Therefore, the levels were taken as per guidelines from Farooq et al. [9] and trial experiments. Similarly, Ahmed et al. [27] used polarity as variable during machining in kerosene dielectric to control material removal mechanism. A thorough analysis of the literature [2,15], early experiments, cost, and time were the foundation for choosing the number of experiments for the second phase. Taguchi’s L_16_ orthogonal array was used to conduct experiments at the levels mentioned in Table 3.

The workpiece was placed on a flat surface during the experimentation to avoid any dimensional error due to an unbalanced position. The tool was grasped firmly in the tool holder. A separate electrode was used for each experiment so that the surface morphology could be monitored easily. Taylor Hobson surface texture meter (UK) was used to measure the surface roughness in terms of arithmetic mean Ra, the highest peak-to-valley distance Rt, and average peak-to-valley distance Rz, retaining the evaluation length at 4 mm and the cut-off length at 0.8 mm. The roughness was measured at five distinct locations in the machined cavity, and the resulting mean values were presented. The machining limitations, such as craters, microcracks, redeposited debris, and surface roughness, were explored using Quanta 450 field emission gun (FEG) scanning electron microscopy. In addition, a pin-on-disc wear tribometer (CSEM Instruments) was used for tribological analysis. The pin-on-disc was used to investigate the friction and wear properties of the machined samples in dry sliding conditions. A milled base plate (EN31 steel disk (60 HRC)) with dimensions of 165 mm diameter and 8 mm thickness with a relatively smooth surface Ra of 0.5 µm (ground after each experiment) was employed as a counter body for tribological assessment. The counter-body surface was ground using SiC-based emery paper of P800 to P1500 grit sizes. After that, a METCO, BAINPOLVTD, Model No. PMV 023 polishing machine was used to achieve Ra 0.5 µm. The cylindrical pin of Ti6Al4V, having 10 mm diameter, was machined at 8 A pulse current to generate the texture for friction testing. The pulse OFF time and ON time were both kept at 75 µs. The pin and disc were cleaned in an acetone bath to eliminate the effect of contaminants. Tests of 1500 s were carried out at room temperature, having 100 N load and 6.28 m/s speed. The surfaces of the discs were prepared at constant machining parameters to ensure the durability of experiments. Tests for machined surfaces of Ti6Al4V with aluminum, brass, copper, and graphite electrodes were considered, and the friction force was plotted against time.

## 3. Process Modeling

**Figure 2:**
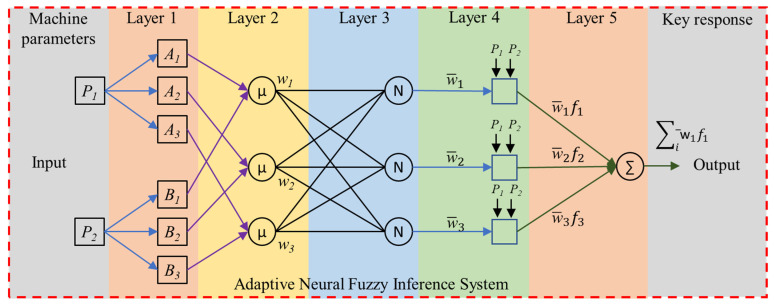
Adaptive neural fuzzy inference system architecture.

Electric discharge machining is a stochastic thermoelectrical material removal process that depends upon different properties of the tool electrodes, workpiece materials, and dielectric system. The current study used a SiC-mixed dielectric to facilitate the erosion process. The modeling and analysis of the system were carried out using the neuro-fuzzy method. This method integrates the inferential abilities of the fuzzy system (FIS) and the data learning capabilities of the artificial neural network (ANN). The adaptive neural fuzzy inference system (ANFIS) uses a sample-based learning approach on the experimental data generated from the parametric settings and develops a structure for efficient prediction [14]. The model based on the ANFIS structure is validated based on test data. In the current study, the L_16_ design of experiments was repeated four times and divided into 75/25 rules for train/test data. The architecture of ANFIS is shown in Figure 2, with two inputs and three membership functions. The critical response as output is one. The machine parameters are key variables that significantly control the particular response. For instance, in the current study, five different variables were chosen.

Layer 1 comprises input membership functions for fuzzification. Each input is processed with membership functions such as triangular, Gaussian, and trapezoidal functions, resulting in the fuzzy membership values *µA_i_(P_1_)* and *µB_i_(P_2_)*. Layer 2 defines the fuzzy rules and calculates the strength (*w_i_*). The rule’s output is processed, and the result is its particular weight. The output is computed based on the input signals, as shown in Equation (1).

(1) $$w_{i}=µA_{i}\left(P_{1}\right)\times µB_{i}\left(P_{2}\right),i=1,2,3$$

Layer 3 normalized the strengths (*w_i_*) as represented in Equation (2).

(2) $${\overset{-}{w}}_{i}=\frac{w_{i}}{\sum_{i}w_{i}},i=1,2,3$$

Similar to layer 2, in which fuzzification is carried out, layer 3 carries out defuzzification. Equation (3) is used to calculate the output of each node.

(3) $${\overset{-}{w}}_{i}.f_{1}={\overset{-}{w}}_{i}\left(a_{i}P_{1}+b_{i}P_{2}+c_{i}\right),i=1,2,3$$

The *a_i_, b_i_*, and *c_i_* values are the consequent parameter sets. Layer 5 calculates the output with one node only, as shown in Equation (4).

(4) $$Output=\sum_{i}{\overset{-}{w}}_{i}.f_{1}$$

The ANFIS parametric settings are shown in Table 4. The Gaussian membership function has better performance with the linear output function than with the constant function. The no. of membership functions was kept to 2 for each parametric input to minimize the computational time by keeping the epoch maximum limit at 30. The testing phase resulted in a maximum error of 0.107 µm.

**Figure 3:**
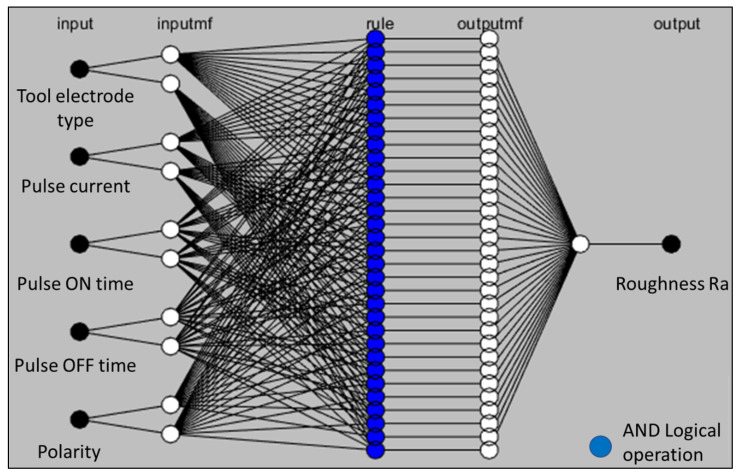
ANFIS architecture for electric discharge machining process.

The complete ANFIS architecture of the electric discharge machining process is shown in Figure 3.

The ANFIS approach uses process input data and develops a prediction model based on training and testing. In the input layer, a comprehensive list of parametric conditions such as tool electrode type, pulse current, pulse ON time, pulse OFF time, and polarity is used with wide ranges of roughening levels. The output layer shows the resulting roughness. The data are not necessarily recommended to comply with any specific design of experiments. Therefore, precise and realistic prediction results are achieved without compromising on any objective. Predictive values are validated using the mean absolute percentage error (MAPE), as is evident in Equation (5).

(5) $$MAPE=\frac{1}{n}\sum_{i=1}^{n}\left|\frac{{Exp}_{i}-{Pred}_{i}}{{Exp}_{i}}\right|,\;n=number\;of\;experiments$$

**Figure 4:**
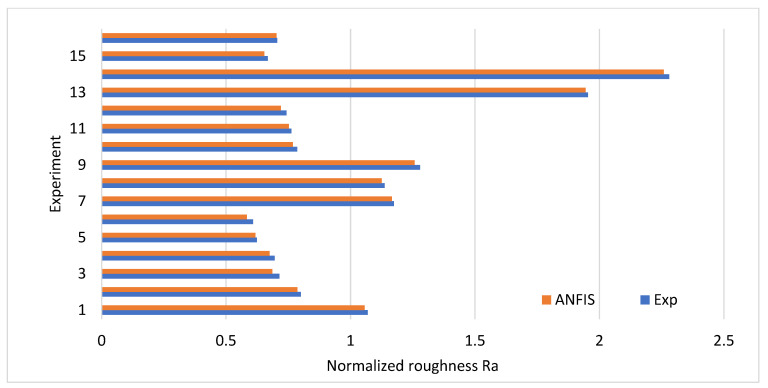
Experimental vs. predicted results.

The hyperparameter tuning of the inference system during training was carried out based on the MAPE, and the lowest error-producing architecture was chosen. The results show a 1.78% MAPE, which is significantly lower than 5%. The normalized roughness Ra results are compared in Figure 4. The predicted results are in close agreement with the experimental results.

## 4. Results and Discussion

### 4.1. Parametric Control Analysis

**Figure 5:**
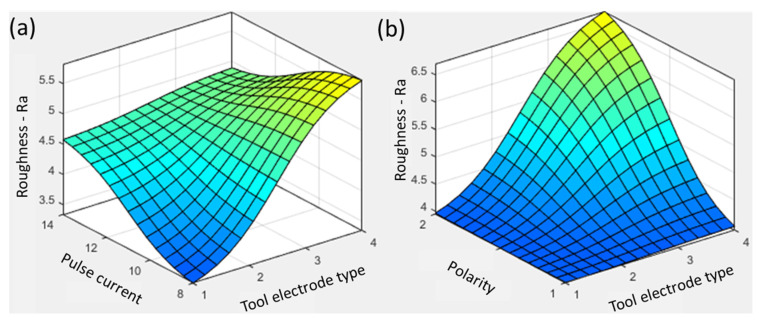
Parametric trends for surface roughness with tool electrode type and (**a**) pulse current and (**b**) polarity.

**Figure 6:**
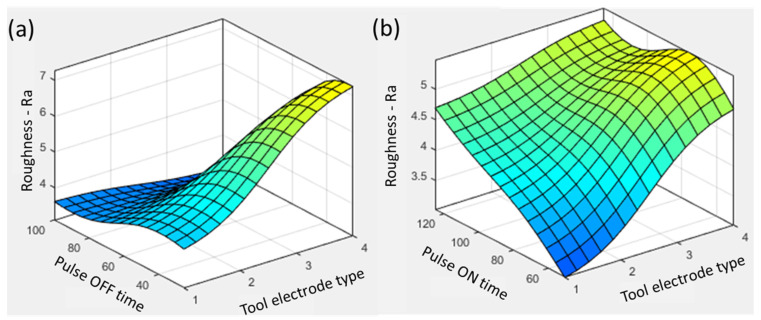
Parametric trends for surface roughness with tool electrode type and (**a**) pulse ON time and (**b**) pulse OFF time.

The parametric effects on the roughness are characterized with two-way interaction plots, as shown in Figure 5 and Figure 6. It is evident in Figure 5a that the graphite resulted in a rougher surface (5.5 µm) compared with the other electrode materials. Graphite has a high melting point (3300 °C), which is the fundamental rationale for high roughness considering the thermoelectric physics of the electric discharge machining process. Ahmed et al. [27] supported similar process science during the machining of Ti6Al4V using conventional dielectric kerosene and found graphite to be the highest roughness-producing electrode material. The material erosion mechanism involves thermal energy production through an electric sparking process when the tool electrode is brought to a significantly small distance from the workpiece surface. The high thermal energy penetrates deep into the workpiece surface, leaving rough craters [29]. Besides tool materials, the pulse current is acknowledged as a significant contributing factor towards the amount of thermal energy on a surface. At low pulse current values (8 A), a low amount of thermal energy is available because of the less intense spark conditions at the interface of the workpiece and tool. Therefore, the low current conditions observed with the aluminum electrodes produced less roughness, with a value of 3.4 µm. With the increase in pulse current to 14 A, the roughness increased to ~4.6 µm, showing a 33% increase using the aluminum electrode. Intense sparking produces high thermal energy, melting and vaporizing the workpiece material [30]. In addition, the tool’s polarity plays a significant role in determining the prime material for erosion. If the tool material’s melting temperature is significantly lower compared with that of the workpiece, then the tool will erode intensely instead of the targeted surface. In this process, shallow craters are formed, reducing the surface roughness [15]. The tool having a higher temperature than the workpiece results in severe surface defects because of the intense energy transfer. Graphite has a 3300 °C melting temperature, whereas the Ti6Al4V workpiece had a melting temperature of 1604–1660 °C, which resulted in the workpiece surface being eroded more and developing deep craters. In the surface plot (see Figure 5a), the copper and brass were placed in the center, resulting in mild roughness at all pulse current levels. Conclusively, the aluminum electrode at a low pulse current (8 A) obtained a lower roughness compared with the graphite electrode.

In both interactive plots in Figure 6, the tool materials show similar trends along with pulse ON and OFF time. The increase in pulse ON time from 50 µs to 125 µs for the graphite tool increased the roughness from ~4.5 µm to ~5.3 µm, showing a 17% rise. The increase in ON time enhanced the energy transfer because of increased sparking time and resulted in the melting of more material on the surface. The thermal conductivity of Ti6Al4V (6.7 W-m^−1^K^−1^) did not sufficiently help with the dissipation of heat, resulting in the deep penetration of sparks and the formation of a rough surface. The increase in pulse ON time from 50 µs to 75 µs resulted in a rougher surface with the highest Ra, 5.5 µm, as shown in Figure 6b, because of the discharge energy transfer and accumulation of heat energy for a longer time period. However, a further increase in pulse ON time resulted in a slight decrease in roughness to ~5.3 µm because of the promotion of an arcing phenomenon during the spark generation against the long pulse duration [28]. During arcing, the discharge column significantly expands because of a longer pulse duration. In this regard, the discharge energy intensity and its amount in the interaction zone decrease on the discharge spot [31]. This phenomenon promotes the formation of shallow craters, resulting in low roughness values. However, in the cases of aluminum, brass, and copper, the effect of arcing is not significant because of their thermal and electrical properties. On the other hand, the rise in pulse OFF time from 25 µs to 100 µs resulted in a decrease in roughness from ~7.1 µm to ~3.3 µm, showing a 53% reduction.

**Figure 7:**
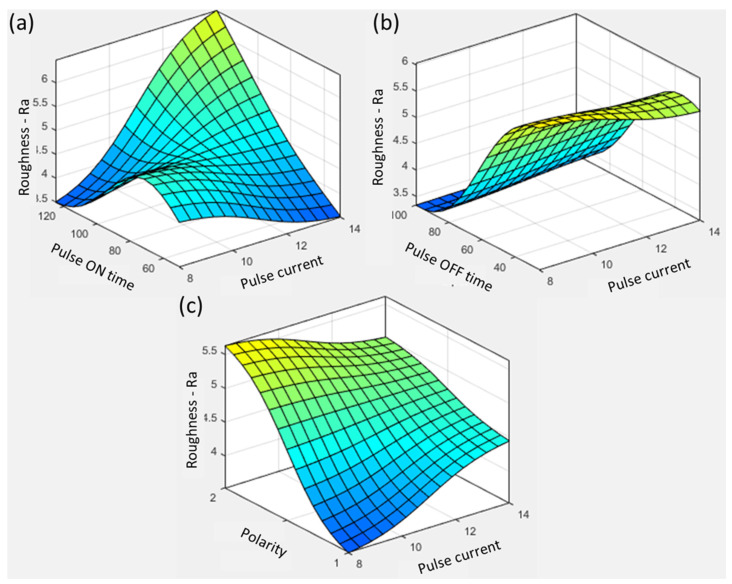
Parametric trends for surface roughness with pulse current and (**a**) pulse ON time, (**b**) pulse OFF time, and (**c**) polarity.

The interactive effect of the pulse current on the other variables is shown in Figure 7. The effect of arcing is visible in Figure 7a, in which roughness decreased because of a further increase in pulse ON time from 75 µs at an 8 A pulse current. With the increase in pulse current from 8 A to 14 A, the spark intensity increased, and removed the arcing produced because of the pulse duration. The highest roughness value, ~6.5 µm, was observed at both extremes of the 14 A pulse current and 125 µs pulse ON time. The trend in pulse OFF time in Figure 7b shows the effectiveness of the flushing attributes. A high pulse current resulted in a poor surface finish at a low pulse OFF time. The higher current introduced increased surface discharge energy, making deep craters and more melt volume. The flushing time to remove the melt was not sufficiently synchronized, resulting in the melt’s redeposition on the surface with altered properties. This recast layer had poor electrical and thermal properties because of carbides generated from the decomposition of the dielectric [32]. Therefore, a balanced flushing duration with high sparking intensity is required to remove material efficiently without creating surface defects. The above discussion concludes that a low pulse ON time with a balanced OFF time is important in order to achieve a high surface finish.

The joint effect of polarity and pulse current on roughness is shown in Figure 7c. The increase in pulse current from 8 A to 14 A with reverse polarity resulted in a significant increase in roughness from ~3.5 µm to ~4.5 µm, showing a 28.5% increase. However, the trend is revised in the case of positive polarity. At all pulse current levels, positive polarity resulted in a rougher surface compared with reverse polarity. At an 8 A pulse current and positive polarity, a roughness of ~5.6 µm was achieved, compared with reverse polarity, which resulted in a roughness of ~3.5 µm.

**Figure 8:**
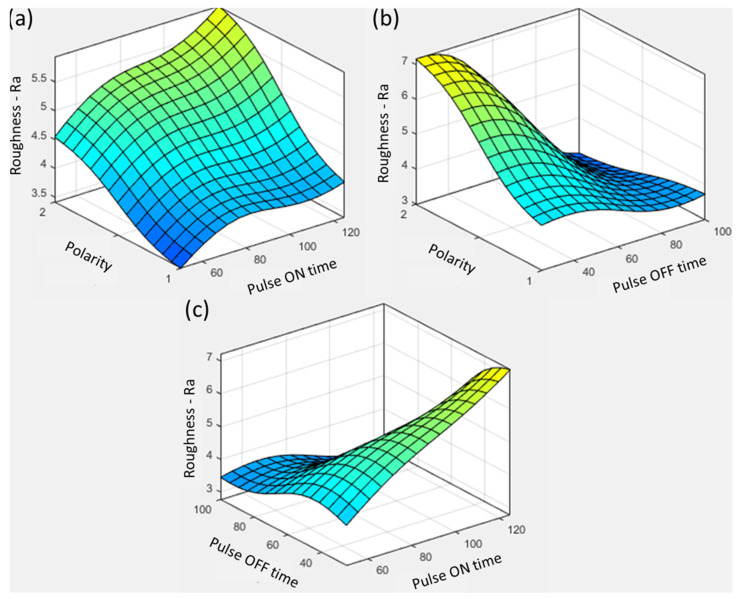
Parametric trends for surface roughness with pulse current and (**a**) pulse ON time, (**b**) pulse OFF time, and (**c**) polarity.

The parametric trends of polarity with other variables are shown in Figure 8. For all pulse ON time values, the reverse polarity has a reduced roughness range compared with positive polarity, as shown in Figure 8a. With the increase in pulse ON time, the roughness is increased because of high-energy transfer to the surface and insufficient thermal conduction in the tool materials as a heat sink. Therefore, the plasma channel expands longer, producing material erosion. Sharma et al. [14] machined Inconel 625 using kerosene as a dielectric and copper as a tool material. The authors found pulse ON and OFF time to be the most influential variables controlling surface roughness. The authors showed correlations of high discharge energy associated with pulse current and ON time with the formation of larger-dimensioned craters. Similar results are shown in Figure 8a, which displays that high thermal energy transfer was primarily responsible for rougher surfaces and intense energy input. With the increase in pulse OFF time, the influence of heat energy was reduced with effective flushing. Low pulse OFF time and positive polarity produced the roughest surface, with Ra = ~7 µm.

The magnitude of influence of pulse ON time is shown in comparison with pulse OFF time (see Figure 8c). As is evident from the above discussion, the amount of discharge energy is closely linked with the workpiece’s surface quality. The graphite produced high roughness values in all conditions because of its melting point at 3300 °C. The copper and brass produced comparable surfaces owing to their thermal conductivities and melting points (copper, 940 °C; brass, 1083 °C). In the case of pulse ON time, the arcing effect was dominant beyond a threshold with restricted enhanced energy transfer, as shown in Figure 8a. The roughness was reduced with a decrease in pulse current associated with the discharge energy and melt pool. Therefore, a synergistic approach is important in establishing surface quality. Low roughness was obtained using positive polarity, 120 µs pulse ON time, 100 µs pulse OFF time, an aluminum electrode, and an 8 A pulse current, based on graphical compromise details.

### 4.2. Surface Evolution with Aluminum

**Figure 9:**
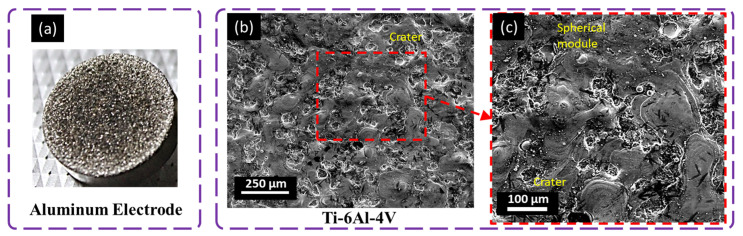
Microscopical analysis of machined surface with an aluminum electrode at 8 A pulse current, 50 µs pulse ON time, 25 µs pulse OFF time, and + polarity; (**a**) tool electrode, (**b**,**c**) workpiece surface.

A comprehensive analysis of the surface morphology of the machined surface was carried out using a scanning electron microscope (SEM). The surface machined with the aluminum electrode (Figure 9) possessed small, shallow, and interconnected craters due to the difference in thermal conductivity between the electrode (205 W-m^−1^K^−1^) and the workpiece (6.7 W-m^−1^K^−1^). The shallowness of the craters was associated with the low energy transfer to the workpiece compared with the electrode. Furthermore, voids and redeposited debris were more prominent on the machined workpiece, owing to its comparable properties (poor heat dissipation) (see Figure 9).

The aluminum electrode has better thermal expansion compared with the workpiece. The factor causing it to erode faster is the debris deposited on the surface at cooling. The poor thermal conductivity of titanium alloy results in it resisting material erosion from a workpiece that causes a greater density of small cracks [33]. Due to the higher surface tension of the melted pool, a spherical module is also observed in microscopic images (Figure 9). Small discrete craters are observed on the electrode tool due to the lower packing density of the atoms on the surface and the lower melting temperature. Discharge energy impacted the tool surface more due to the melting temperature and thermal conductivity difference that modified the morphology of the workpiece surface in terms of cracks, spherical modules, and redeposited debris [2]. Similarly, Hascalik et al. [34] reported that fewer cracks, pores, and craters were developed during the machining of titanium alloy compared with copper and graphite electrodes in a comparative study.

### 4.3. Surface Evolution with Brass

**Figure 10:**
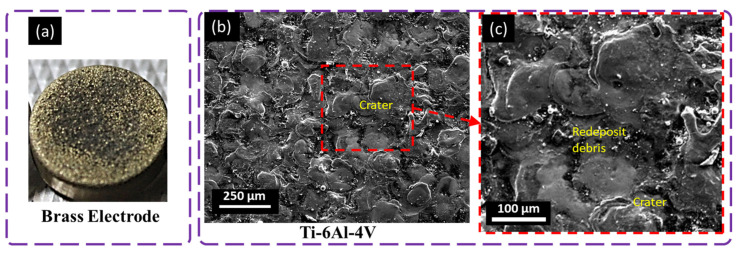
Microscopical analysis of machined surface with brass electrode at 8 A pulse current, 100 µs pulse ON time, 100 µs pulse OFF time, and + polarity; (**a**) tool electrode, (**b**,**c**) workpiece surface.

Figure 10 shows that the brass tool resulted in a different surface morphology compared with all the other selected electrodes and machined surfaces of titanium alloy with variable characteristics. The machined surface with the brass electrode possessed a higher number of craters and cracks than that with the copper electrode. Higher surface roughness was observed on the surface machined with Br than that machined with copper in terms of Ra and Rt, which were 5.45% and 36.36%, respectively. Bhaumik et al. [5] determined that Br has a lower thermal conductivity that causes it to develop a spark for a long time and erode more material compared with copper electrodes, with a higher heat dissipation rate during the machining of titanium alloy. The workpiece electrode showed greater and deeper craters at specific positions. This is because of the poor thermal conductivity of the brass electrode (Figure 10).

The impulsive and concentrated discharge at a specific position eroded the workpiece surface and increased the MRR; however, this led to deeper craters on the workpiece surface, and the same erosion was prominent on the tool interface. Micropores were observed on the surface due to discharge concentration, flushing, and the removal of sucked air bubbles which cause microporosity on the workpiece as well as on the tool interface, so the surface finishing was compromised in the case of machining with a brass electrode due to the irregular machining pattern [35]. Senthilkumar et al. [36] evaluated that more residual stresses are developed on the machined surface during the pulse ON and OFF time for the extended machining time of brass electrodes compared with copper electrodes for the machining of metal matrix composites.

### 4.4. Surface Evolution with Copper

**Figure 11:**
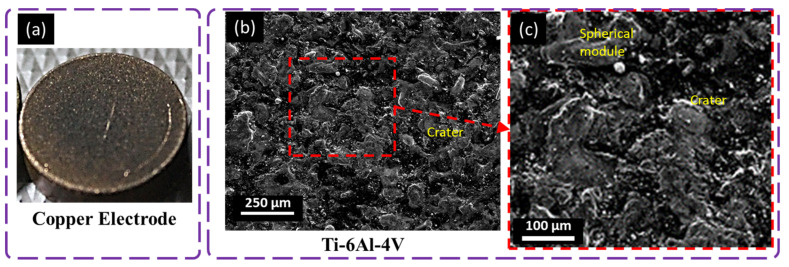
Microscopical analysis of machined surface with a copper electrode at 8 A pulse current, 75 µs pulse ON time, 75 µs pulse OFF time, and − polarity; (**a**) tool electrode, (**b**,**c**) workpiece surface.

Small, interconnected craters, microcracks, spherical modules, melted drops, and globules of debris are examined in the SEM micrographs of the titanium surface machined with the copper electrode tool in Figure 11. Copper is known for its high thermal and electrical conductivity, volumetric expansion, and structural integrity. The copper tool was used because discharge is concentrated and distributed over the tool interface. This phenomenon causes a large number of craters to appear on the machined surface. Melted drops and globules of debris formed due to the copper tool’s high volumetric expansion, inhibiting proper flushing and debris accumulation on the surface (Figure 11). Due to the melting pool’s high tension, spherical modules were formed on the machined titanium alloy [37]. The copper electrodes generated more air bubbles and a thick recast layer with many cracks, voids, and redeposited pieces of debris compared with the graphite electrodes due to the copper electrodes’ high thermal conductivity and less sintered nature. Chen et al. [38] investigated a similar process, in which a copper electrode was used to machine titanium alloy (Ti–6A1–4V) in relatively less intense conditions.

### 4.5. Surface Evolution with Graphite

**Figure 12:**
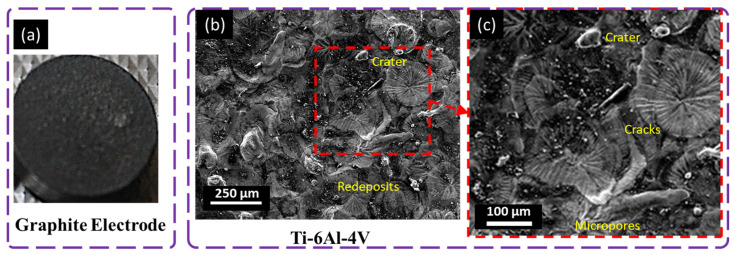
Microscopical analysis of machined surface with graphite electrode at 8 A pulse current, 125 µs pulse ON time, 50 µs pulse OFF time, and − polarity; (**a**) tool electrode, (**b**,**c**) workpiece surface.

The SEM micrograph in Figure 12 shows the surface morphology of the titanium alloy machined with the graphite electrode. The machined surface contains large, deeper craters and globules of debris. A proposed reason for this is the higher melting temperature, at 3300 °C, of the graphite electrode than that of the titanium alloy at 1660 °C, owing to higher melting temperature discharge energy putting more impact on the electrode with the lowest melting point. Due to this, a thick recast layer was generated on the workpiece surface, but with fewer microvoids. Over the cooling and flushing periods, debris globules were attached to the workpiece surface. Micropores and voids are observed less on the workpiece surface because graphite is a sintered compact material. Carbon black was embedded in the workpiece surface during machining, reducing its porosity (Figure 12).

From the interface of the graphite electrode, it can also be inferred that much less tool wear was observed due to the small and compact particles of the graphite electrode and the high melting point, and the overlapping of carbon content that ultimately reduced the tool wear. The surface machined with the graphite electrode showed the highest surface roughness of all those machined with the selected electrodes, with an appropriate material removal rate. In contrast, aluminum showed the lowest surface roughness and material removal rate [39]. This process science is in line with Lee and Li [40], who evaluated the impact of three electrodes: copper–tungsten, graphite, and copper. The graphite electrode machining showed higher MRR, higher surface roughness, and fewer cracks on the tool electrode surface than the copper and copper–tungsten electrodes due to its high thermal conductivity and melting point.

### 4.6. Tribological Analysis of Machined Surfaces

**Figure 13:**
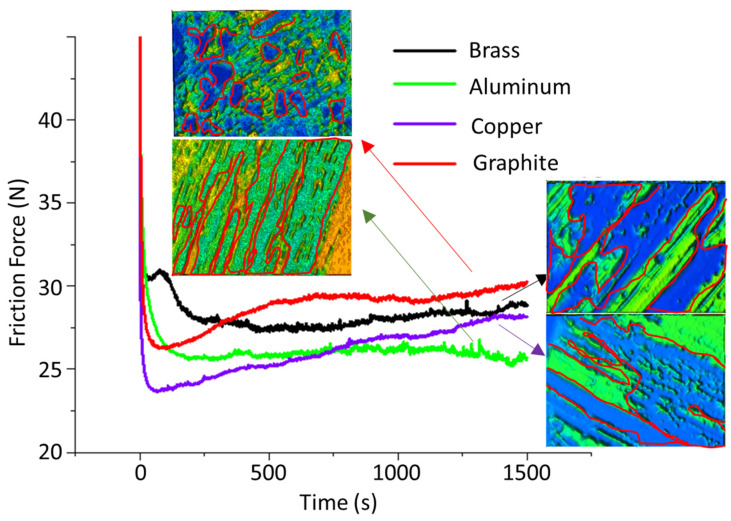
Microscopical analysis of machined surface with graphite electrode at 8 A pulse current, 125 µs pulse ON time, 50 µs pulse OFF time, and − polarity.

The tribological tests characterized the friction force between the Ti6Al4V machined surface and the reference material. At the start of the test, the peaks and valleys were smoothened, which resulted in a higher friction force. However, the force decreased with time and stabilized at a certain level. The particular behavior and stabilized values are used for comparison, and the color-contrasted surface micrographs at the end were used for wear mechanism analysis. In Figure 13, micrographs are presented with wear scars marked with red lines. The light blue color shows the intensity of the wear mechanisms.

The surface machined with graphite had deep craters where the friction force suddenly decreased at the start (Figure 12) due to plastic deformation and smoothening of the surface. The wear tracks are highlighted in the micrograph and the deep craters are enveloped in red. The friction force at the end of the cycle was ~30 N. Deep sliding marks were found for the surface generated with aluminum, and the surface was smoothened with time. At the end of the cycle, it showed a minimum friction force of ~25.5 N compared with others. Moreover, the brass (~28 N) and copper (~27.5 N) resulted in similar magnitudes at the end of the test and comparable surface properties. A smoothening effect was experienced at a higher roughness, which also affirmed the higher friction force (25 N to 30 N). The wear tracks show scratches and shallow craters along with the plastic flow and severe deformation in terms of abrasion. A similar wear mechanism is observed in the literature during the tribological characterization of electric discharge machined surfaces. For instance, Adnan et al. [35] compared friction forces under different roughness conditions on AISI 304L steel, with the result that friction forces (1.5 N to 6.5 N) were higher for surfaces having high roughness, which are prone to being deformed because of smoothening against a 20 N load. The electric discharged machined surface has a (random-crater-patterned) recast layer of significantly higher hardness than the base material. Mughal et al. [8] carried out a detailed chemical-based surface characterization that showed the material transfer from the tool electrode, workpiece, dielectric, and powder (SiC) during the machining of Ti6Al4V. The authors discussed the improvement in surface hardness. Therefore, the current study used SiC powder to improve the surface’s hardness and control the surface defects, contributing to its tribological performance. The joint effect of roughness and hardness in the case of EDMed surfaces influences the wear performance. A similar understanding is discussed by Usman et al. [41] with the support of Archard’s law of abrasive wear. This law explains the mechanistic degradation of surfaces as follows: W = kps/H, where W = total volume of produced wear, s = sliding distance, p = normal pressure, k = dimensionless wear coefficient, and H = surface hardness. On the other hand, Philip et al. [42] evaluated different heat treatments on Ti6Al4V and carried out tribological testing. The friction force was observed to vary from 30 to 50 N against a 0 to 200 N load. As per these conclusions, the authors recommended better alloy performance against a 100 N load and commented on the improvement in wear performance because of heat treatment and surface chemistry. The electric discharge machining process significantly improved the surface properties, resulting in superior tribological performance. The wear mechanisms were found to be in line with the literature.

## 5. Conclusions

This study was carried out to investigate the potentiality of electric discharge machining of Ti6Al4V for tribological applications in the biomedical industry. A wide range of process parameters, including four types of electrodes, were evaluated to quantify their influence over different surface parameters. Based on the thorough experimental results, the following conclusions are drawn:

1. With the increase in pulse current to 14 A, the roughness increased to ~4.6 µm, showing a 33% increase using the aluminum electrode.
2. The increase in pulse ON time from 50 µs to 125 µs for the graphite tool resulted in a roughness increase from ~4.5 µm to ~5.3 µm, showing a 17% rise. The increase in ON time enhances the energy transfer because of increased sparking time and melts more material on the surface.
3. The surface machined through the aluminum electrode possessed small, shallow, and interconnected craters.
4. Copper electrodes generated more air bubbles and a recast layer with many cracks, voids, and redeposited pieces of debris compared with graphite electrodes due to the copper electrodes’ high thermal conductivity and density.
5. The machined surface contains large, deeper craters and globules of debris. The origin of these features is the higher melting temperature, at 3300 °C, of the graphite electrode than that of the titanium alloy at 1660 °C.
6. The hyperparameter tuning of the inference system during training was carried out based on the MAPE. The lowest error-producing architecture was chosen, resulting in a 1.78% MAPE, significantly less than 5%.
7. A smoothening effect was experienced at higher roughness values (such as was the case with the graphite electrode), which also affirmed higher friction forces (generally ranging from 5.8 N to 8 N).
8. The wear tracks showed scratches and shallow craters along with the plastic flow and severe deformation in terms of abrasion.

## Figures and Tables

**Table 1:** Physical and mechanical properties of workpiece material (Open Access Webpage [26]).

Properties	Value
Hardness (Vicker D)	320
Density (g/cm^3^)	4.43
Yield strength (MPa)	955
Ultimate tensile strength (MPa)	990
Modulus of elasticity (GPa)	114
Thermal conductivity (W-m^−1^K^−1^)	6.7
Electrical resistivity (µΩ-cm)	178
Melting point (°C)	1660

**Table 2:** Physical and mechanical properties of workpiece material (Open Access [27]).

Tool Material	Thermophysical Properties			
	Density (g-cm^−3^)	Melting Point (°C)	Thermal Conductivity (W-m^−1^K^−1^)	Electrical Conductivity (S-m^−1^)
Graphite	320	3300	400	0.3 × 10^6^
Copper	4.43	1083	385	59.6 × 10^6^
Brass	955	940	109	16 × 10^6^
Aluminum	1660	660	205	35 × 10^6^

**Table 3:** Parametric conditions with levels for experimentation.

Sr. No	Parameter	Units	Levels			
			1	2	3	4
1	Tool electrode type	-	Aluminum (Al)	Copper (Cu)	Brass (Br)	Graphite (Gr)
2	Pulse current	A	8	10	12	14
3	Pulse ON time	µs	50	75	100	125
4	Pulse OFF time	µs	25	50	75	100
5	Polarity	-	Reverse	Positive		
6	Dielectric	-	Kerosene oil + 5 g/L SiC			

**Table 4:** Parametric conditions for ANFIS.

Training Method	MF Type	Output Function	No. MFs	Epoch	Avg. Test Error	MAPE
Hybrid	Gaussian	Linear	2 2 2 2 2	30	0.107 µm	1.78%

## Data Availability

The data are already available in the manuscript.

## References

[1] Xie Z.J., Mai Y.J., Lian W.Q., He S.L., Jie X.H. (2016). Titanium Carbide Coating with Enhanced Tribological Properties Obtained by EDC Using Partially Sintered Titanium Electrodes and Graphite Powder Mixed Dielectric. Surf. Coat. Technol..

[2] Farooq M.U., Ali M.A., He Y., Khan A.M., Pruncu C.I., Kashif M., Ahmed N., Asif N. (2020). Curved Profiles Machining of Ti6Al4V Alloy through WEDM: Investigations on Geometrical Errors. J. Mater. Res. Technol..

[3] Farooq M.U., Anwar S. (2023). Investigations on the Surface Integrity of Ti6Al4V under Modified Dielectric(s)-Based Electric Discharge Machining Using Cryogenically Treated Electrodes. Processes.

[4] Asif N., Saleem M.Q., Farooq M.U. (2023). Performance Evaluation of Surfactant Mixed Dielectric and Process Optimization for Electrical Discharge Machining of Titanium Alloy Ti6Al4V. CIRP J. Manuf. Sci. Technol..

[5] Bhaumik M., Maity K. (2018). Effect of Different Tool Materials during EDM Performance of Titanium Grade 6 Alloy. Eng. Sci. Technol. Int. J..

[6] Farooq M.U., Bhatti H.A., Asad M., Kumar M.S., Zahoor S., Khan A.M. (2022). Surface Generation on Titanium Alloy through Powder-Mixed Electric Discharge Machining with the Focus on Bioimplant Applications. Int. J. Adv. Manuf. Technol..

[7] Dong H., Bell T. (2000). Enhanced Wear Resistance of Titanium Surfaces by a New Thermal Oxidation Treatment. Wear.

[8] Mughal M.P., Farooq M.U., Mumtaz J., Mia M., Shareef M., Javed M., Jamil M., Pruncu C.I. (2021). Surface Modification for Osseointegration of Ti6Al4V ELI Using Powder Mixed Sinking EDM. J. Mech. Behav. Biomed. Mater..

[9] Umar Farooq M., Pervez Mughal M., Ahmed N., Ahmad Mufti N., Al-Ahmari A.M., He Y. (2020). On the Investigation of Surface Integrity of Ti6Al4V ELI Using Si-Mixed Electric Discharge Machining. Materials.

[10] Al-Amin M., Abdul-Rani A.M., Rao T.V.V.L.N., Danish M., Rubaiee S., bin Mahfouz A., Parameswari R.P., Wani M.F. (2022). Investigation of Machining and Modified Surface Features of 316L Steel through Novel Hybrid of HA/CNT Added-EDM Process. Mater. Chem. Phys..

[11] Ahmed W., Jackson M.J. (2016). Surgical Tools and Medical Devices.

[12] Ehsan S., Rehman M., Mughal M.P., Farooq M.U., Ali M.A. (2022). Machinability Investigations through Novel Controlled Flushing Characteristics in Wire Electric Discharge Machining of M42 High-Speed Steel. Int. J. Adv. Manuf. Technol..

[13] Rafaqat M., Mufti N.A., Ahmed N., Rehman A.U., AlFaify A.Y., Farooq M.U., Saleh M. (2022). Hole-Making in D2-Grade Steel Tool by Electric-Discharge Machining through Non-Conventional Electrodes. Processes.

[14] Sharma D., Bhowmick A., Goyal A. (2022). Enhancing EDM Performance Characteristics of Inconel 625 Superalloy Using Response Surface Methodology and ANFIS Integrated Approach. CIRP J. Manuf. Sci. Technol..

[15] Ishfaq K., Farooq M.U., Pruncu C.I. (2021). Reducing the Geometrical Machining Errors Incurred during Die Repair and Maintenance through Electric Discharge Machining (EDM). Int. J. Adv. Manuf. Technol..

[16] Tang L., Du Y.T. (2014). Experimental Study on Green Electrical Discharge Machining in Tap Water of Ti-6Al-4V and Parameters Optimization. Int. J. Adv. Manuf. Technol..

[17] Hassanin H., Modica F., El-Sayed M.A., Liu J., Essa K. (2016). Manufacturing of Ti-6Al-4V Micro-Implantable Parts Using Hybrid Selective Laser Melting and Micro-Electrical Discharge Machining. Adv. Eng. Mater..

[18] Tiwary A.P., Pradhan B.B., Bhattacharyya B. (2018). Investigation on the Effect of Dielectrics during Micro-Electro-Discharge Machining of Ti-6Al-4V. Int. J. Adv. Manuf. Technol..

[19] Sultan T., Kumar A., Gupta R.D. (2014). Material Removal Rate, Electrode Wear Rate, and Surface Roughness Evaluation in Die Sinking EDM with Hollow Tool through Response Surface Methodology. Int. J. Manuf. Eng..

[20] Dong H., Liu Y., Li M., Zhou Y., Liu T., Li D., Sun Q., Zhang Y., Ji R. (2019). Sustainable Electrical Discharge Machining Using Water in Oil Nanoemulsion. J. Manuf. Process..

[21] Payal H.S., Choudhary R., Singh S. (2008). Analysis of Electro Discharge Machined Surfaces of EN-31 Tool Steel. JSIR.

[22] Thoe T.B., Aspinwall D.K., Wise M.L.H., Oxley I.A. (1996). Polycrystalline Diamond Edge Quality and Surface Integrity Following Electrical Discharge Grinding. J. Mater. Process. Technol..

[23] Rajurkar K.P., Levy G., Malshe A., Sundaram M.M., McGeough J., Hu X., Resnick R., DeSilva A. (2006). Micro and Nano Machining by Electro-Physical and Chemical Processes. CIRP Ann..

[24] Bui V.D., Mwangi J.W., Schubert A. (2019). Powder Mixed Electrical Discharge Machining for Antibacterial Coating on Titanium Implant Surfaces. J. Manuf. Process..

[25] Li L., Zhao L., Li Z.Y., Feng L., Bai X. (2017). Surface Characteristics of Ti-6Al-4V by SiC Abrasive-Mixed EDM with Magnetic Stirring. Mater. Manuf. Process..

[26] Material Science|News|Materials Engineering|News. https://www.azom.com/.

[27] Ahmed N., Anwar S., Ishfaq K., Rafaqat M., Saleh M., Ahmad S. (2019). The Potentiality of Sinking EDM for Micro-Impressions on Ti-6Al-4V: Keeping the Geometrical Errors (Axial and Radial) and Other Machining Measures (Tool Erosion and Work Roughness) at Minimum. Sci. Rep..

[28] Raza S., Kishore H., Nirala C.K., Rajurkar K.P. (2023). Multiphysics Modelling and High-Speed Imaging-Based Validation of Discharge Plasma in Micro-EDM. CIRP J. Manuf. Sci. Technol..

[29] Li L., Hou R.G., SiMa Z.W. (2012). Electrical Discharge Machining of Nickel-Based Super Alloy. Advanced Materials Research.

[30] Papazoglou E.L., Karmiris-Obratański P., Leszczyńska-Madej B., Markopoulos A.P. (2021). A Study on Electrical Discharge Machining of Titanium Grade2 with Experimental and Theoretical Analysis. Sci. Rep..

[31] Kumar D., Sisodiya M.S., Mandal D.K., Bajpai V. (2023). Maglev Micro-EDM: Feasibility and Performance on Inconel 625. CIRP J. Manuf. Sci. Technol..

[32] Selvarajan L., Rajavel R., Venkataramanan K., Srinivasan V.P. (2023). Experimental Investigation on Surface Morphology and Recasting Layer of Si_3_N_4_-TiN Composites Machined by Die-Sinking and Rotary EDM. Ceram. Int..

[33] Mughal K., Mughal M.P., Farooq M.U., Anwar S., Ammarullah M.I. (2023). Using Nano-Fluids Minimum Quantity Lubrication (NF-MQL) to Improve Tool Wear Characteristics for Efficient Machining of CFRP/Ti6Al4V Aeronautical Structural Composite. Processes.

[34] Hasçalık A., Çaydaş U. (2007). A Comparative Study of Surface Integrity of Ti-6Al-4V Alloy Machined by EDM and AECG. J. Mater. Process. Technol..

[35] Czelusniak T., Higa C.F., Torres R.D., Laurindo C.A.H., de Paiva Júnior J.M.F., Lohrengel A., Amorim F.L. (2019). Materials Used for Sinking EDM Electrodes: A Review. J. Braz. Soc. Mech. Sci. Eng..

[36] Senthilkumar V., Omprakash B.U. (2011). Effect of Titanium Carbide Particle Addition in the Aluminium Composite on EDM Process Parameters. J. Manuf. Process..

[37] Kolli M., Adepu K. (2014). Influence of Span 20 Surfactant and Graphite Powder Added in Dielectric Fluid on EDM of Titanium Alloy. Bonfring Int. J. Ind. Eng. Manag. Sci..

[38] Chen S.L., Yan B.H., Huang F.Y. (1999). Influence of Kerosene and Distilled Water as Dielectrics on the Electric Discharge Machining Characteristics of Ti-6A1-4V. J. Mater. Process. Technol..

[39] Muttamara A., Borwornkiatkaew W., Pronpijit A., Nuanchom S. (2016). Effect of Graphite Electrode to Surface’s Characteristic of EDM. MATEC Web of Conferences.

[40] Lee S.H., Li X.P. (2001). Study of the Effect of Machining Parameters on the Machining Characteristics in Electrical Discharge Machining of Tungsten Carbide. J. Mater. Process. Technol..

[41] Usman M., Ishfaq K., Rehan M., Raza A., Mumtaz J. (2023). An In-Depth Evaluation of Surface Characteristics and Key Machining Responses in WEDM of Aerospace Alloy under Varying Electric Discharge Environments. Int. J. Adv. Manuf. Technol..

[42] Philip J.T., Kumar D., Mathew J., Kuriachen B. (2020). Experimental Investigations on the Tribological Performance of Electric Discharge Alloyed Ti-6Al-4V at 200–600 °C. J. Tribol..

